# The Aesthetically Ideal Position of the Nipple–Areola Complex on the Breast

**DOI:** 10.1007/s00266-016-0684-z

**Published:** 2016-08-05

**Authors:** Richard Lewin, Matteo Amoroso, Nikolina Plate, Clara Trogen, Gennaro Selvaggi

**Affiliations:** Department of Plastic Surgery, Institute of Clinical Sciences, Sahlgrenska Academy, Sahlgrenska University Hospital, University of Gothenburg, Gröna Stråket 8, 41345 Gothenburg, Sweden

**Keywords:** Aesthetic breast, Breast surgery, Nipple position on the breast

## Abstract

**Background:**

Several studies have attempted to identify an objective description of the aesthetically ideal breast, but they have all suffered in their reliability because of having several intrinsic limitations. It is therefore essential to design a template of ideal breast features in order to predict and evaluate aesthetic outcomes in both reconstructive and cosmetic breast surgery. The aim of this study was to determine the aesthetically preferred position of the nipple–areola complex on the breast.

**Methods:**

A questionnaire was sent by regular mail to 1000 men and 1000 women aged between 16 and 74 years. They were asked to rank the attractiveness of a series of breasts of women in images with different NAC positions. The images showed breasts from two different angles: 12 frontal-view images with both breasts shown, and five side-view images with only one breast shown. All of the breasts had equal dimensions and proportions, with the same areola size but different NAC positions. Statistical analysis of data was carried out.

**Results:**

Eight hundred and thirteen of 2000 participants completed the questionnaire. The NAC placement preferred by both genders had a ratio of 40:60 *x* and 50:50 *y*, which means that it was best situated in the middle of the breast gland vertically and slightly lateral to the midpoint horizontally. Significant differences were found between the age and gender subgroup preferences.

**Conclusions:**

This study identified the preferred position of the nipple–areola complex on the female breast in the general population. This is an important information when planning breast reconstructive and cosmetic surgery.

**Level of Evidence II:**

This journal requires that authors assign a level of evidence to each article. For a full description of these Evidence-Based Medicine ratings, please refer to the Table of Contents or the online Instructions to Authors www.springer.com/00266.

## Introduction

Surgery of the breast is a broad field of plastic surgery. Regardless of the nature of the procedure, whether reconstructive or cosmetic, the main goal is always to recreate a natural appearance of the breast and to meet the patient’s expectations [1]. The correspondence between physical appearance and psychological body image is an important aspect of a patient’s quality of life after breast surgery [1, 2] and the study of this relationship requires an appropriate aesthetic outcome assessment [3–5]. Several studies have attempted to identify an objective description of the aesthetically ideal breast, but they have all suffered from shortcomings in their reliability, because of having several intrinsic limitations [6–12] such as the small number of subjects investigated, a lack of measurable results, and an untrustworthy study design. As a consequence, aesthetic evaluation of the breast is still poorly defined [13]. It is therefore essential to design a template of ideal breast features to predict and evaluate aesthetic outcomes in both reconstructive breast surgery and cosmetic breast surgery. Many of these features are very subjective, and no one can really say that large breasts are aesthetically superior to small breasts. As we all know, beauty is in the eye of the beholder. But the location of the nipple–areola complex (NAC) is of great importance even if the other characteristics such as size and shape differ, i.e. hanging breasts look more odd if the NAC is displaced—as would large breasts without ptosis. The placement of the nipple–areola complex has a significant effect on the overall appearance of a woman’s breast [11].

The purpose of this study was to identify, using the general population, the most aesthetically favourable position of the nipple–areola complex on the female breast.

## Methods

We designed a randomized cross-sectional questionnaire study. A questionnaire was sent by regular mail to 1000 men and 1000 women aged between 16 and 74 years, who were randomly chosen from a government database of the entire Swedish population. In this way, we obtained opinions from a large number of participants over a limited period of time. They were asked to rank the attractiveness of a series of breasts of women in images with different NAC positions. The questionnaire was designed to be anonymous in order to obtain higher response rates and more truthful answers and, consequently, unbiased data [14]_._ Furthermore, to avoid selection bias related to the order of the images, we used a random sequence generator [15] to create 4 different variants of the questionnaire provided. This would create 4 different groups per gender with 250 individuals in each.

### Design of the Questionnaire

The questionnaire was designed to determine the most favoured placement of the nipple–areola complex on the female breast using the responses to the questionnaire. In the first part of the questionnaire, the participants were asked to give their age and gender (Questions 1 and 2). The second part was a series of frontal and lateral profile images of female breasts with different NAC positions.

**Fig. 1:**
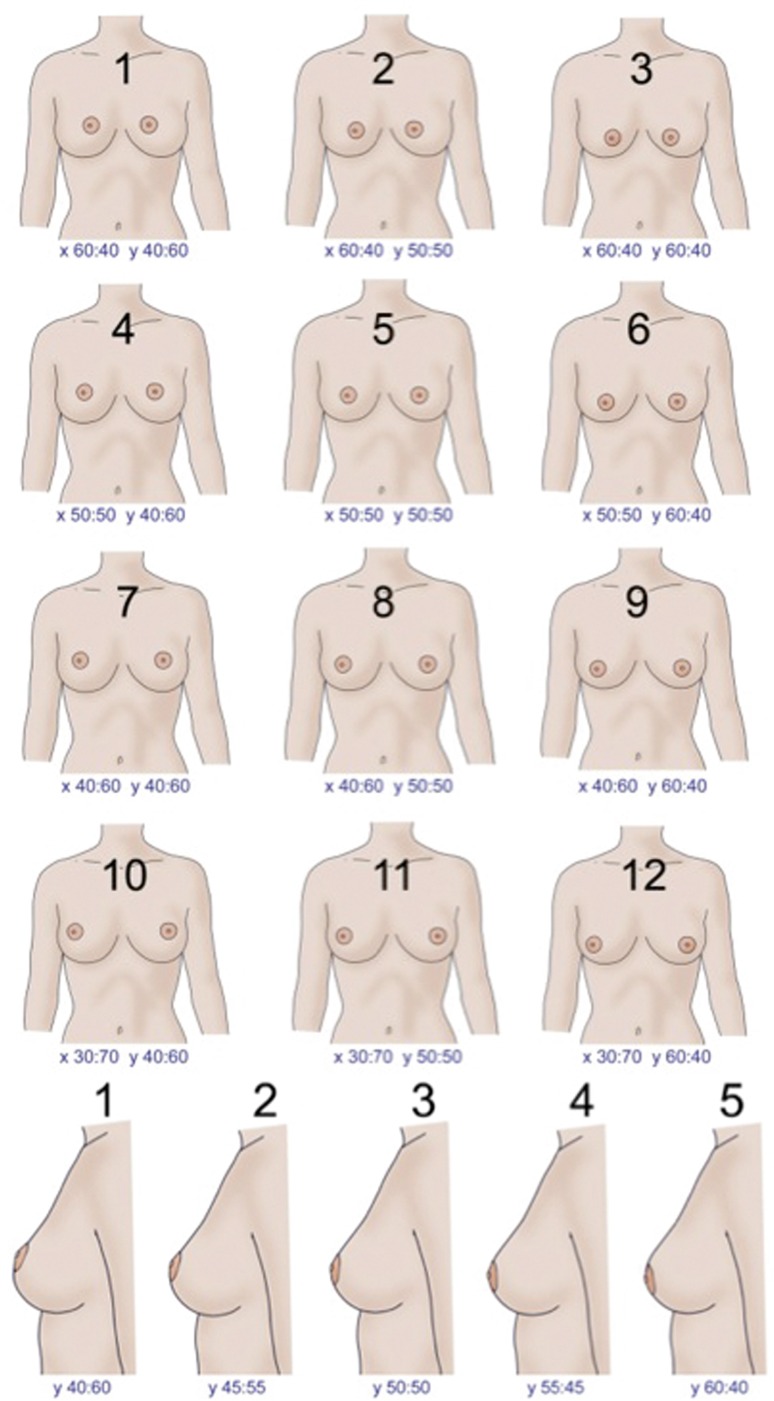
The 12 frontal images and the 5 profile images, with associated *x*- and *y*-ratios , respectively

The images showed breasts from two different angles: 12 frontal-view images with both breasts shown, and five side-view images with only one breast shown (Fig. 1). All the breasts had equal dimensions and proportions between upper pole slope and lower pole convexity (upper pole–lower pole ratio), with the same areola size but different NAC positions. This method allowed us to objectively determine the preferences of participants independently of the size and proportions of the breasts, and solely based on NAC position (which was the only varying factor).

The participants were asked to rank their three favourite frontal images, grading from 1 to 3, with number 1 being the most favourite, number 2 the second best, and number 3 the third best. From the side-view images, they were asked to select only the most pleasing one (Questions 3 and 4).

### Positioning of the Nipple–Areola Complex and Breast Geometry

To place the NAC in the most appropriate position, we developed a method based on breast anatomy and mathematical considerations [16–20]. Several studies based on algebraic formulae have defined the breast as a complex geometrical structure [16–19]. In particular, a recent advanced geometric calculation defined the breast as a complex paraboloid structure, with two half paraboloids sharing the same elliptical base, and having different focus for the upper and lower halves of the breast along the virtual plane [20]. The nipple sits at this upper/lower pole boundary (the nipple meridian) [11].

**Fig. 2:**
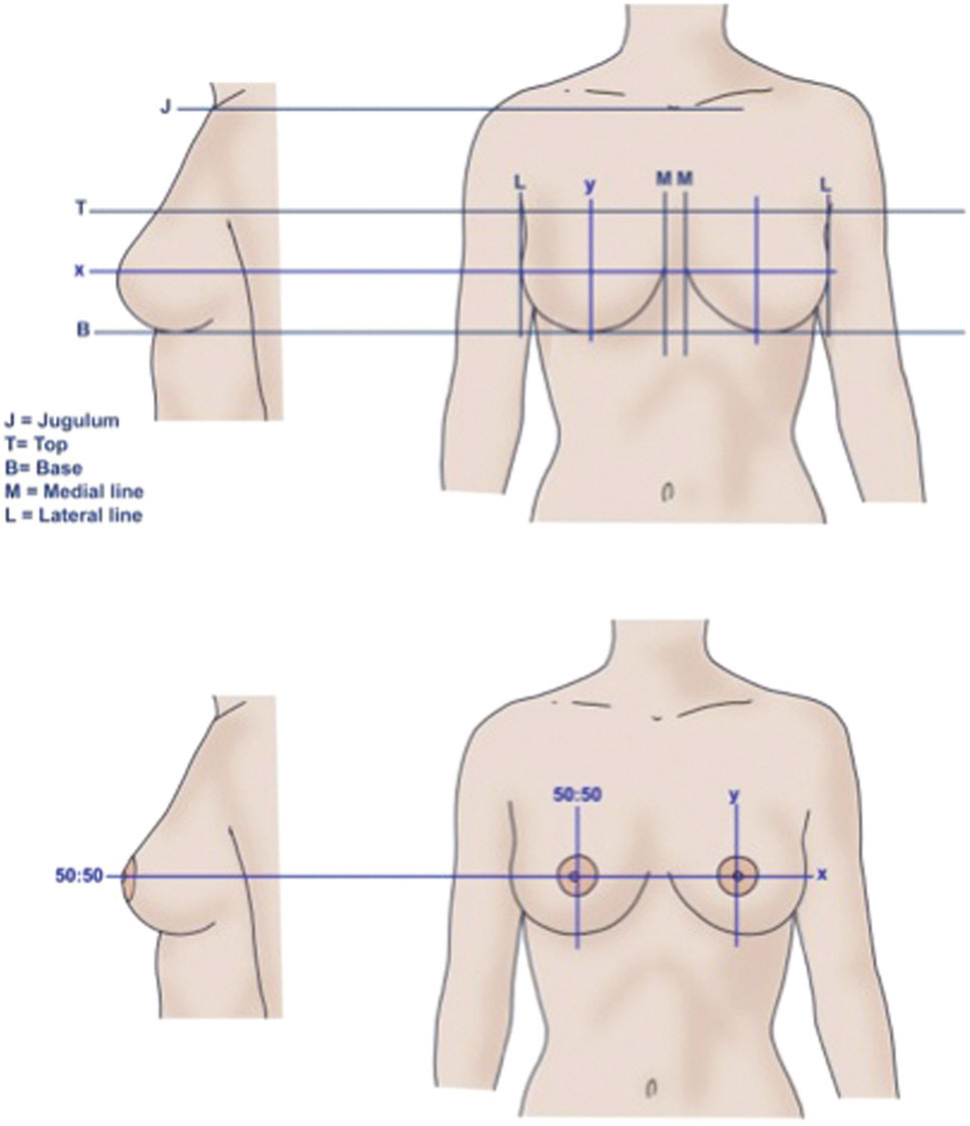
The outlines of the breast gland. *Upper panels* the coordinate system of the breast. *Lower panels* an example of NAC placement in a 50:50 *x*- and *y*-ratio, both *horizontally* and *vertically*

In our work, we compared frontal images and profile images. We outlined the breast with a medial line, a lateral line, an upper line, and a lower line (Fig. 2, upper panel). Using the ruler function in Photoshop CS5, we divided the breast with a horizontal and a vertical axis (*x*- and *y*-axes). The midpoint of the two axes corresponded to the midpoint of the breast gland. With this coordinate system, we moved the NAC both horizontally and vertically, and in this way various breast appearances were obtained (Fig. 2, lower panel).

### Statistical Analysis

Statistical analysis of data was carried out using IBM SPSS Statistics version 22.0. Using Fisher’s exact test for dichotomous variables, we compared the responses from female and male participants to determine gender-related preferences. The Mantel–Haenszel Chi-Square exact test was used to study the relationship between breast preferences and age. The Chi-Square exact test was chosen for non-ordered categorical variables, to determine whether the placement of the images influenced the answers.

## Results

### Respondent Demographics

Eight hundred and thirteen of 2000 participants completed the questionnaire (350 men and 463 women). The largest proportion of responders were in the age group 61–75 years [*n* = 229 (28 %)]. The group aged 16–30 [*n* = 144 (18 %)] and the group aged 31–40 [*n* = 144 (18 %)] had an identical proportion of responders, which was also the lowest. The average age of responders was 48.0 years. Demographic data are summarized in Table 1.

**Table 1:** Demographic data

Variable	Total (*n* = 813)	Men (*n* = 350)	Women (*n* = 463)
Age	47.9 (16.0) 48.0 (16.0; 75.0)	47.7 (16.0) 48.0 (16.0; 75.0)	48.0 (16.0) 49.0 (16.0; 75.0)
Age group			
16–30	144 (18 %)	63 (18.0 %)	81 (17.5 %)
31–40	144 (18 %)	60 (17 %)	84 (18 %)
41–50	151 (19 %)	69 (20 %)	82 (18 %)
51–60	145 (18 %)	66 (19 %)	79 (17 %)
61–75	229 (28 %)	92 (26 %)	137 (30 %)

For categorical variables, data are *n* (%). For continuous variables, data are mean (SD) followed by median (min; max)

### Results by Gender

**Fig. 3:**
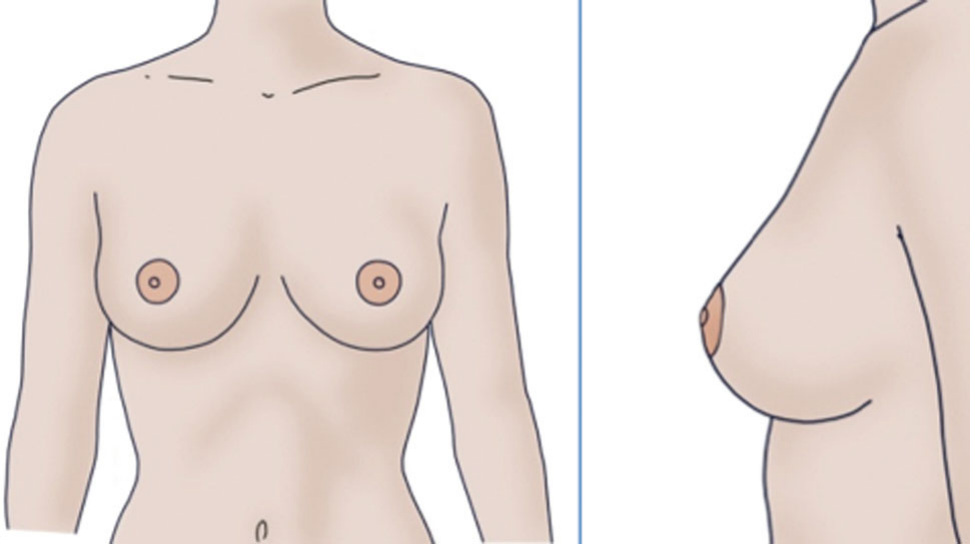
Regardless of the gender of the responders, frontal image number 8 (*left panel*) (with a ratio of 40:60 *x* 50:50 *y*) was the frontal image most often selected. Image number 3 (ratio 50:50 *y*) was the profile image most often selected (*right panel*)

Frontal image number 8 (ratio of 40:60 *x*; 50:50 *y*) (Fig. 3, left panel) was the most frequently chosen image regardless of the gender of the responder [30.4 % (*n* = 211) 95 % CI 27.0–33.9]. Frontal images number 7 (ratio of 40:60 *x*; 40:60 *y*) and 9 (ratio of 40:60 *x*; 60:40 *y*) were the second [19 % (*n* = 132) 95 % CI 16.1–22.1] and third [17.1 % (*n* = 117) 95 % CI 14.4–20.1] most frequently chosen images (Table 2).

**Table 2:** First choice of frontal and profile images, according to gender

Variable	Total (*n* = 815)	Men (*n* = 352)	Women (*n* = 463)	*P*-value
Frontal images				
Frontal image 1	2 (0.3 %)	0 (0.0 %)	2 (0.5 %)	ns
Frontal image 2	1 (0.1 %)	1 (0.3 %)	0 (0.0 %)	ns
Frontal image 3	1 (0.1 %)	0 (0.0 %)	1 (0.3 %)	ns
Frontal image 4	26 (3.7 %)	8 (2.6 %)	18 (4.6 %)	ns
Frontal image 5	36 (5.2 %)	14 (4.6 %)	22 (5.6 %)	ns
Frontal image 6	32 (4.6 %)	10 (3.3 %)	22 (5.6 %)	ns
Frontal image 7	132 (19.0 %)	62 (20.4 %)	70 (17.9 %)	ns
Frontal image 8	211 (30.4 %)	85 (28.0 %)	126 (32.2 %)	ns
Frontal image 9	119 (17.1 %)	45 (14.8 %)	74 (18.9 %)	ns
Frontal image 10	30 (4.3 %)	19 (6.3 %)	11 (2.8 %)	0.044
Frontal image 11	68 (9.8 %)	40 (13.2 %)	28 (7.2 %)	0.012
Frontal image 12	37 (5.3 %)	20 (6.6 %)	17 (4.3 %)	ns
Profile images				
Profile image 1	64 (7.9 %)	27 (7.7 %)	37 (8.0 %)	ns
Profile image 2	196 (24.1 %)	98 (28.0 %)	98 (21.2 %)	0.030
Profile image 3	338 (41.6 %)	154 (44.0 %)	184 (39.7 %)	ns
Profile image 4	159 (19.6 %)	52 (14.9 %)	107 (23.1 %)	0.0040
Profile image 5	56 (6.9 %)	19 (5.4 %)	37 (8.0 %)	ns

For categorical variables, data are *n* (%). For comparisons between groups, Fisher’s exact test was used for dichotomous variables

However, although the most frequently chosen frontal image was the same for both men and women (frontal image number 8), differences between men and women were found concerning the frontal images selected as second and third choices, but this was not statistically significant (*P* > 0.05) (Table 2).

**Fig. 4:**
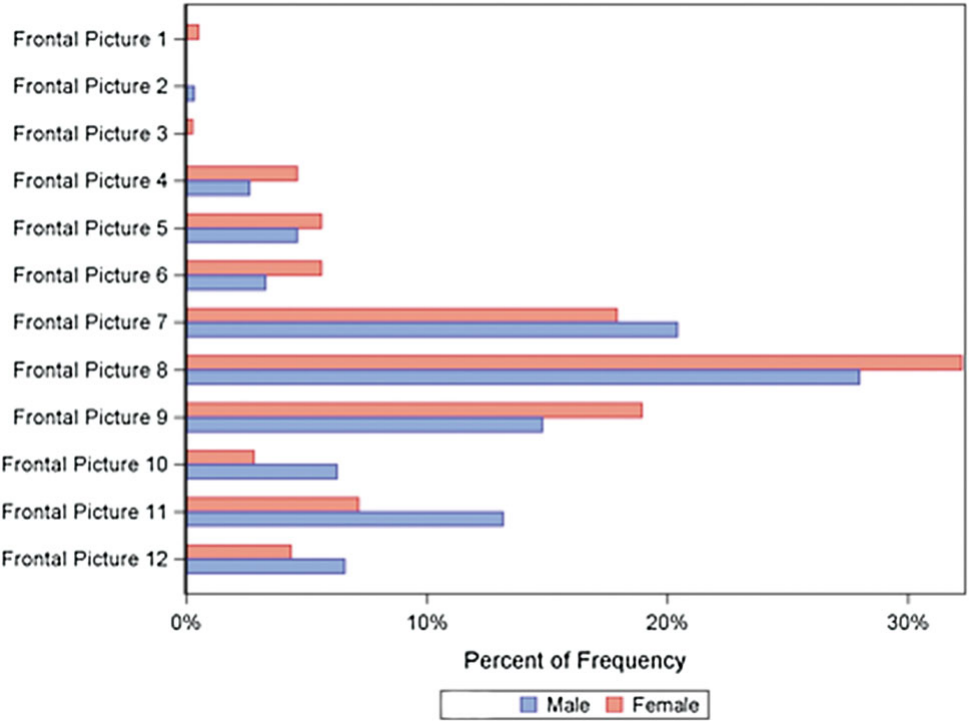
Choice of frontal image according to gender, including the first 3 chosen

A significantly greater proportion of men [13.2 % (*n* = 40) 95 % CI 9.6–17.5] than women [7.2 % (*n* = 28) 95 % CI 4.8–10.2] ranked frontal image number 11 (ratio of 30:70 *x*; 50:50 *y*) as their preferred type of breast (*P* < 0.05) (Table 2). Moreover, there was a statistically significant difference between men and women in the overall choice of three frontal images (Table 3). For example, 65.8 % of men (*n* = 200) (95 % CI 60.2–71.1) selected frontal image number 8 (ratio of 40:60 *x*; 50:50 *y*) among their three best choices, as compared to 76.2 % (*n* = 298) (95 % CI 71.7–80.4) of women. This difference was statistically significant (*P* < 0.05) (Table 3). Although a significantly greater proportion of men selected frontal image number 10 (ratio of 30:70 *x*; 40:60 *y*) among their three best choices (95 % CI 17.5–27.1; *P* < 0.001), a significantly greater proportion of women selected frontal images number 5, 6, and 9 (95 % CI 25.7–35.0, 15.9–24.0, and 46.8–57.0, respectively; *P* < 0.05) (Table 3 ; Fig. 4).

**Table 3:** First, second, and third choice of frontal image, according to gender

Variable	Total (*n* = 815)	Men (*n* = 352)	Women (*n* = 463)	*P*-value
Frontal images				
Frontal image 1	8 (1.2 %)	5 (1.6 %)	3 (0.8 %)	ns
Frontal image 2	6 (0.9 %)	4 (1.3 %)	2 (0.5 %)	ns
Frontal image 3	10 (1.4 %)	8 (2.6 %)	2 (0.5 %)	0.044
Frontal image 4	109 (15.7 %)	52 (17.1 %)	57 (14.6 %)	ns
Frontal image 5	184 (26.5 %)	66 (21.7 %)	118 (30.2 %)	0.015
Frontal image 6	111 (16.0 %)	34 (11.2 %)	77 (19.7 %)	0.0030
Frontal image 7	299 (43.0 %)	130 (42.8 %)	169 (43.2 %)	ns
Frontal image 8	498 (71.7 %)	200 (65.8 %)	298 (76.2 %)	0.0033
Frontal image 9	333 (47.9 %)	130 (42.8 %)	203 (51.9 %)	0.020
Frontal image 10	112 (16.1 %)	67 (22.0 %)	45 (11.5 %)	0.0003
Frontal image 11	230 (33.1 %)	106 (34.9 %)	124 (31.7 %)	ns
Frontal image 12	151 (21.7 %)	76 (25.0 %)	75 (19.2 %)	ns

For categorical variables, data are *n* (%). For comparison between groups, Fisher’s exact test was used for dichotomous variables

Regarding the profile image results, profile image number 3 (ratio 50:50 *y*) (Fig. 3, right panel) was the most frequently chosen image [41.6 % (*n* = 338) 95 % CI 38.2–45.0], followed by profile image number 2 (ratio 45:55 *y*) and profile image number 4 (ratio 55:45 *y*) as the second [24.1 % (*n* = 196) 95 % CI 21.2–27.2] and third [19.6 % (*n* = 159) 95 % CI 16.9–22.5] most frequently chosen images, respectively, regardless of the gender of the individuals interviewed (Table 2). These data correspond to the results for the frontal images.

However, although the most frequently chosen profile image was the same for both men and women (profile image number 3), statistically significant differences were found between the profile images selected as second and third choices (*P* < 0.05).

**Fig. 5:**
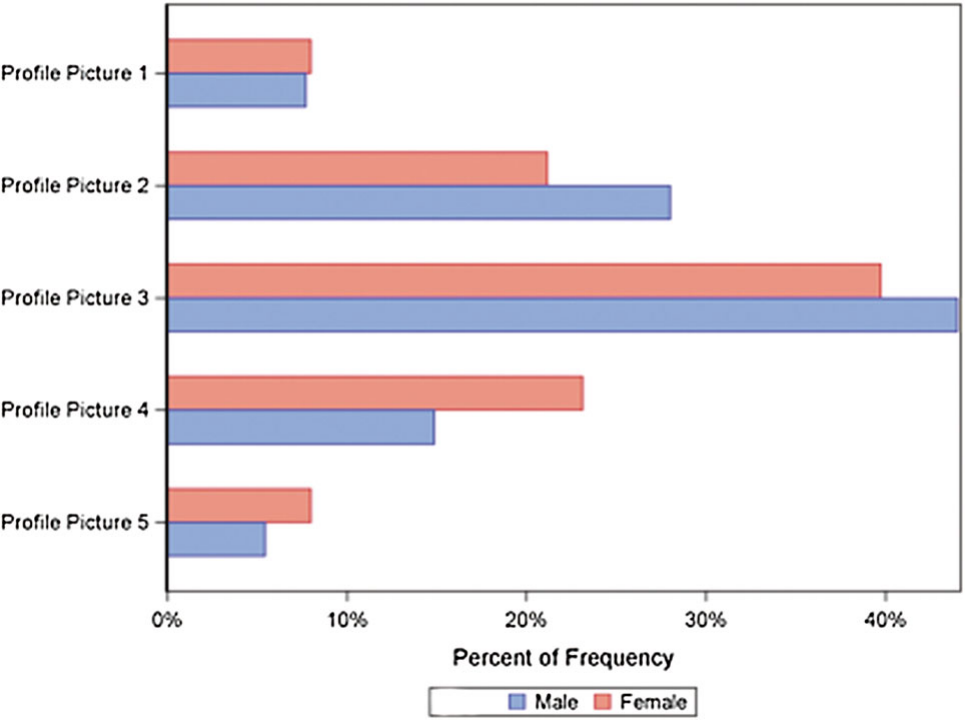
Choice of profile image according to gender

A statistically significant difference in the profile image most frequently chosen by men and women was also found (Table 2). Although a significantly greater proportion of men [28 % (*n* = 98)] than women [21.2 % (*n* = 98)] ranked profile image number 2 as the most attractive (*P* < 0.05), a significantly greater proportion of women [23.1 % (*n* = 107)] than men [14.9 % (*n* = 52)] selected profile image number 4 instead (*P* < 0.05) (Table 2 ; Fig. 5).

### Results According to Age

We compared the images that were chosen most frequently by responders who were less than 35 years old and those who were 35 years old or more, of both genders (Table 4).

**Table 4:** First choice of frontal and profile images, according to gender and age group: overall test and pairwise between groups

Variable	Females aged 16–34 years (*n* = 117)	Females aged ≥35 years (*n* = 346)	Males aged 16–34 years (*n* = 88)	Males ≥35 (*n* = 264)	*P*-value	Test between groups *P*-value					
						Females aged 16–34 years vs. females aged ≥ 35 years	Females aged 16–34 years vs. males aged 16–34 years	Females aged 16–34 years vs. males aged ≥35 years	Females aged ≥35 years vs. males aged 16–34 years	Females aged ≥35 years vs. males aged ≥35 years	Males aged 16–34 years vs. males aged ≥35 years
Frontal images											
Frontal image 1	0 (0.0 %)	2 (0.7 %)	0 (0.0 %)	0 (0.0 %)	ns	ns	ns	ns	ns	ns	ns
Frontal image 2	0 (0.0 %)	0 (0.0 %)	0 (0.0 %)	1 (0.4 %)	ns	ns	ns	ns	ns	ns	ns
Frontal image 3	0 (0.0 %)	1 (0.3 %)	0 (0.0 %)	0 (0.0 %)	ns	ns	ns	ns	ns	ns	ns
Frontal image 4	4 (4.0 %)	14 (4.8 %)	2 (2.6 %)	6 (2.7 %)	ns	ns	ns	ns	ns	ns	ns
Frontal image 5	3 (3.0 %)	19 (6.6 %)	0 (0.0 %)	14 (6.2 %)	ns	ns	ns	ns	0.019	ns	0.028
Frontal image 6	4 (4.0 %)	18 (6.2 %)	1 (1.3 %)	9 (4.0 %)	ns	ns	ns	ns	ns	ns	ns
Frontal image 7	13 (12.9 %)	57 (19.7 %)	12 (15.4 %)	50 (22.1 %)	ns	ns	ns	ns	ns	ns	ns
Frontal image 8	36 (35.6 %)	90 (31.0 %)	19 (24.4 %)	66 (29.2 %)	ns	ns	ns	ns	ns	ns	ns
Frontal image 9	15 (14.9 %)	59 (20.3 %)	18 (23.1 %)	27 (11.9 %)	0.033	ns	ns	ns	ns	0.014	0.032
Frontal image 10	4 (4.0 %)	7 (2.4 %)	8 (10.3 %)	11 (4.9 %)	0.023	ns	ns	ns	0.010	ns	ns
Frontal image 11	15 (14.9 %)	13 (4.5 %)	12 (15.4 %)	28 (12.4 %)	0.0009	0.0021	ns	ns	0.0036	0.0018	ns
Frontal image 12	7 (6.9 %)	10 (3.4 %)	6 (7.7 %)	14 (6.2 %)	ns	ns	ns	ns	ns	ns	ns
Profile images											
Profile image 1	10 (8.5 %)	27 (7.8 %)	4 (4.6 %)	23 (8.7 %)	ns	ns	ns	ns	ns	ns	ns
Profile image 2	35 (29.9 %)	63 (18.2 %)	24 (27.6 %)	74 (28.1 %)	0.0086	0.013	ns	ns	ns	0.0051	ns
Profile image 3	45 (38.5 %)	139 (40.2 %)	43 (49.4 %)	111 (42.2 %)	ns	ns	ns	ns	ns	ns	ns
Profile image 4	20 (17.1 %)	87 (25.1 %)	12 (13.8 %)	40 (15.2 %)	0.0063	ns	ns	ns	0.029	0.0035	ns
Profile image 5	7 (6.0 %)	30 (8.7 %)	4 (4.6 %)	15 (5.7 %)	ns	ns	ns	ns	ns	ns	ns

For categorical variables, data are *n* (%). For comparison between groups, Chi-Square exact test was used for non-ordered categorical variables. For pairwise comparisons between groups. Fisher’s exact test was used for dichotomous variables

Women less than 35 years of age most often gave frontal image number 8 as their first choice [35.6 % (*n* = 36) 95 % CI 26.4–45.8]. Frontal image number 9 (ratio of 40:60 *x*; 60:40 *y*) and frontal image number 11 (ratio of 30:70 *x*; 50:50 *y*) were the second most frequently chosen images [14.9 % (*n* = 15) 95 % CI 8.6–23.3], followed by frontal image number 7 (ratio of 40:60 *x*; 40:60 *y*) as the third most frequently chosen image [12.9 % (*n* = 13) 95 % CI 7.0–21.0].

Women over 35 years of age most often gave frontal image number 8 as their first choice [31 % (*n* = 90) 95 % CI 25.8–36.7]. Frontal image number 9 (ratio of 40:60 *x*; 60:40 *y*) and frontal image number 7 (ratio of 40:60 *x*; 40:60 *y*) were the second [20.3 % (*n* = 59) 95 % CI 15.9–25.4] and third [19.7 % (n = 57) 95 % CI, 15.2–24.7] most frequently chosen images.

Men less than 35 years of age most often gave frontal image number 8 as their first choice [24.4 % (*n* = 19) 95 % CI 15.3–35.4], followed by frontal image number 9 (ratio of 40:60 *x*; 40:60 *y*) as the second most frequently chosen image [23.1 % (*n* = 18) 95 % CI 14.3–34.0]. Frontal image number 7 (ratio of 40:60 *x*; 40:60 *y*) and frontal image number 11 (ratio of 30:70 *x*; 50:50 *y*) were the third most frequently chosen images [15.4 % (*n* = 12) 95 % CI 8.2–25.3].

Men over 35 years of age most often gave frontal image number 8 as their first choice [29.2 % (*n* = 66) 95 % CI 23.4–35.6]. Frontal image number 7 (ratio of 40:60 *x*; 40:60 *y*) and frontal image number 11 (ratio of 30:70 *x*; 50:50 *y*) were the second [22.1 % (*n* = 50) 95 % CI 16.9–28.1] and third [12.4 % (*n* = 28) 95 % CI 8.4–17.4] most frequently chosen images.

Significant differences were found between the subgroups (Table 4).

A significantly greater proportion of women less than 35 years of age chose frontal image number 11 (ratio of 30:70 *x*; 50:50 *y*) as their favourite frontal image compared to women over 35 years of age (*P* < 0.05).

A significantly greater proportion of men less than 35 years of age most often chose frontal image number 11 (ratio of 30:70 *x*; 50:50 *y*) (*P* < 0.05) or frontal image number 10 (ratio of 30:70 *x*; 40:60 *y*) (*P* < 0.001) as their favourite frontal image compared to women over 35 years of age.

A significantly greater proportion of women over 35 years of age most often chose frontal image number 9 (ratio of 40:60 *x*; 60:40 *y*) as their favourite frontal image compared to men over 35 years of age (*P* < 0.05), who instead most often chose frontal image number 11 (ratio of 30:70 *x*; 50:50 *y*) (*P* < 0.001).

Of the profile images, number 3 (ratio 50:50 *y*) (Fig. 3, right panel) was the image most frequently given as the first choice irrespective of age (Table 4). Women less than 35 years of age and the male population in general most often selected profile image number 2 (ratio 45:55 *y*) and profile image number 4 (ratio 55:45 *y*) as their second and third choices, without any significant differences between them (Table 4). In contrast, women over 35 years of age most often selected profile image number 4 (ratio 55:45 *y*) and profile image number 2 (ratio 45:55 *y*) as their second and third choices (*P* < 0.05).

### Results from the Four Versions of the Survey

As mentioned in the Methods section, to prevent selection bias we distributed four different versions of the questionnaire. No significant differences were found.

## Discussion

Several studies have attempted to establish an objective template for the ideal position of NAC, but the reliability of all of them was questionable because they had several intrinsic design limitations [6–13, 19].

J. Penn discussed dimensions and placement of the nipples and areolas in “an attractive breast” [6]. The most aesthetic nipple location was said to be at the two basal angles of an imaginary equilateral triangle with its apex at the sternal notch and each side measuring 21 cm. However, the small number of subjects investigated (20 women aged between 18 and 39 years) and the evaluation method (based on the author’s preferences) limited the value of this study.

Mallucci and Branford attempted to define a template of the aesthetically ideal female breast [11, 12]. However, the reliability of their results suffered from several limitations in study design. In fact, in their first study (2011) [11], they identified four key parameters (upper pole–lower pole ratio, nipple angulation, upper pole slope, lower pole convexity) to define the aesthetically ideal female breast assuming that the NAC position is always aligned with the level of maximum breast projection. On the contrary, nipple meridian and the level of maximum breast projection do not always coincide. The quantitative assessment was carried out by analysing 4 independent variables simultaneously, whereas these should have been assessed independently. Moreover, the images published by the media often represent models with unnatural postures, and these cannot be considered suitable for scientific purposes. Several other criticisms of Mallucci’s work were also raised by Swanson [21].

Later (2014), the same authors [12] carried out an observational study and evaluated the opinions of 1,315 men and women, including 53 plastic surgeons, on ideal breast proportions. Responders were asked to rank the attractiveness of images of four women with morphed breasts of different proportions, between the upper and lower poles of the breast, in three-quarter profile poses.

For our breast template, we decided to use the same key features as described in the first study by Mallucci and Branford [11]: proportions of the upper and lower poles in a ratio of 45:55, the upper pole slope linear or slightly concave, and the lower pole convex. In contrast to Mallucci and Branford, we defined the placement of the nipple–areola complex in an objective manner. Furthermore, in our questionnaire we used only one image, keeping volume and shape as fixed parameters and with the position of the nipple as the only variable. We preferred to use cartoon line-drawing images of breasts to eliminate other sources of bias such as, for example, NAC size and relationship between NAC site/position and breast size. These elements could affect the responder’s choices.

Frontal image number 8 (ratio of 40:60 *x*; 50:50 *y*) and profile image number 3 (ratio 50:50 *y*) were the most frequently chosen images, regardless of the gender of the responder. This suggests that the position of the NAC should be in the middle of the *y*-axis, and slightly lateral to the meridian of the *x*-axis. The explanation of the preferred 40:60 *x*-ratio may be related to the physiological, slightly lateral position of the breasts due to the concave shape of the chest, which results in a more natural breast appearance.

By looking at the top three and the bottom three choices of men and women, it appears that the nipple placement vertically is more important in the overall evaluation. All of the top three choices of both men and women had the same *x*-ratio (40:60), but the height of the NAC (the *y*-ratio) varied. Similarly, the bottom three choices shared the same *x*-ratio, corresponding to the most medial nipple placement (an *x*-ratio of 60:40).

This can be interpreted as a preference for a lower NAC position (frontal images number 6 and 9) by women, and a preference for a higher-placed NAC by men (frontal image number 10). In addition, the range of preferences of men was much wider whereas the range of preferences of women tended to be more selective (Table 3).

Similarly, profile image results showed that men prefer a nipple placement higher up (45:55 *y*-ratio; number 2) while women prefer a lower position (55:45 *y*-ratio; number 4).

For both men and women, the height of the NAC placement was less important than the medial/lateral position. The reason for these differences might be that men tend to have a more youthful breast in mind and are perhaps also influenced by media such as “The Sun” newspaper compared to women, who in most cases are regularly exposed to nude women—for example, in public baths—and their choice may be more of a realistic one.

Results of age subgroups indicated that younger women and men tended to select breasts with the NAC placed higher up. These results might be, for women, influenced by their own age group who might have less ptotic breasts as a natural breast development, and for men perhaps also from the media and the internet. However, preferences investigated in the study may not coincide with anthropometrical measurements.

This investigation highlights the preferences of the general public, which can depend, for example, on images created by the mass media. Nevertheless, surgeons should not forget anatomical standards, and they should instruct patients on what is realistically achievable by surgery. Even so, this study identified a potential template for the preferred nipple–areola complex placement on the female breast, which may be of use in making meaningful comparisons between surgical techniques and in making advances in treatment. The next step will be to compare our findings with existing postoperative results to use this template as a guide for NAC placement in both reconstructive and cosmetic breast surgery.

## Conclusion

This study determined the preferences for the nipple–areola complex on the female breast in our study population. The NAC placement preferred by both genders had a ratio of 40:60 *x* and 50:50 *y*, which means that it was best situated in the middle of the breast gland vertically and slightly lateral to the midpoint horizontally (Fig. 3). The second most frequently chosen NAC placement by men and young women was 40:60 *x*; 40:60 *y*, and older women’s second most chosen position was 40:60 *x* and 60:40.

## References

[1] Soon Kim M, Sbalchiero CJ, Reece PG, Miller JM, Beahm KE, Markey K (2008). Assessment of breast aesthetics. Plast Reconstr Surg.

[2] Sarwer DB, Cash TF, Magee L (2005). Female college students and cosmetic surgery: an investigation of experiences, attitudes, and body image. Plast Reconstr Surg.

[3] Andrade WN, Semple JL (2006). Patient self-assessment of the cosmetic results of breast reconstruction. Plast Reconstr Surg.

[4] Kovacs L, Zimmermann A, Papadopulos NA (2004). Re: factors determining shape and symmetry in immediate breast reconstruction. Ann Plast Surg.

[5] von Soet T, Kvalem IL, Skolleborg KC (2006). Psychosocial factors predicting the motivation to undergo cosmetic surgery. Plast Reconstr Surg.

[6] Penn J. Breast reduction. Br J Plast Surg. 1955;7(4):357–371. 10.1016/s0007-1226(54)80046-4 13230442

[7] Fabié A, Delay E, Chavoin JP, Soulhiard F, Seguin P (2006). Plastic surgery application in artistic studies of breast cosmetic. Ann Chir Plast Esthet.

[8] Westreich M (1997). Anthropomorphic breast measurement: protocol and results in 50 women with aesthetically perfect breasts and clinical application. Plast Reconstr Surg.

[9] Liu YJ, Thomson JG (2011). Ideal anthropomorphic values of the female breast: correlation of pluralistic aesthetic evaluations with objective measurements. Ann Plast Surg.

[10] Khan HA, Bayat A (2008). A geometric method for nipple localization. Can J Plast Surg.

[11] Mallucci P, Branford OA (2012). Concepts in aesthetic breast dimensions: analysis of the ideal breast. J Plast Reconstr Aesthet Surg.

[12] Mallucci P, Branford OA (2014). Population analysis of the perfect breast: a morphometric analysis. Plast Reconstr Surg.

[13] Brody GS (2004). The perfect breast: is it attainable? does it exist?. Plast Reconstr Surg.

[14] Econometrics of Anonymized Micro Data: Sonderheft 5/2005 Jahrbücher für Nationalökonomie und StatistikSep 2005 by Winfried Pohlmeier and Gerd Ronning

[15] Random Sequence Generator (2015): Dr Mads Haahr; https://www.random.org/sequences/.Accessed 20 Feb 2015

[16] Katariya RN, Forrest AP, Gravelle IH (1974). Breast volumes in cancer of the breast. Br J Cancer.

[17] Kalbhen CL (1999). Mammographic determination of breast volume: comparing different methods. AJR Am J Roentgenol.

[18] Fung, James T (2010). Mammographic determination of breast volume by elliptical cone estimation. World J Surg.

[19] Smith DJ, Palin WE, Katch VL, Bennett JE (1986). Breast volume and anthropomorphic measurements: normalvalues. Plast Reconstr Surg.

[20] Maitra IK, Sanjay N, Bandyopadhyay KS (2012). A computerized approach towards breast volume calculation. Int J Appl Inf Syst.

[21] Swanson E (2015). Ideal breast shape: women prefer convexity and upper pole fullness. Plast Reconstr Surg.

